# A Stabilized Kriging Method for Mapping Disease Rates

**DOI:** 10.2188/jea.JE20210276

**Published:** 2023-04-05

**Authors:** Che-Chia Hsu, Dai-Rong Tsai, Shih-Yung Su, Jing-Rong Jhuang, Chun-Ju Chiang, Ya-Wen Yang, Wen-Chung Lee

**Affiliations:** 1 Institute of Epidemiology and Preventive Medicine, College of Public Health, National Taiwan University, Taipei, Taiwan; 2 Innovation and Policy Center for Population Health and Sustainable Environment, College of Public Health, National Taiwan University, Taipei, Taiwan; 3 Taiwan Cancer Registry, Taipei, Taiwan

**Keywords:** kriging method, incidence, oral cancer, disease map

## Abstract

**Background:**

Mapping disease rates is an important aspect of epidemiological research because it helps inform public health policy. Disease maps are often drawn according to local administrative areas (LAAs), such as counties, cities, or towns. In LAAs with small populations, disease rates are unstable and are prone to appear extremely high or low. The empirical Bayes methods consider variance differences among different LAAs, thereby stabilizing the disease rates. The methods of kriging break the constraints of geopolitical boundaries and produce a smooth curved surface in the form of contour lines, but the methods lack the stabilizing effect of the empirical Bayes methods.

**Methods:**

An easy-to-implement stabilized kriging method is proposed to map disease rates, which allows different errors in different LAAs.

**Results:**

Monte Carlo simulations revealed that the stabilized kriging method had smaller symmetric mean absolute percentage error than three other types of methods (the original LAA-based method, empirical Bayes methods, and traditional kriging methods) in nearly all scenarios considered. Real-world data analysis of oral cancer incidence rates in men from Taiwan demonstrated that the age-standardized rates in the central mountainous sparsely-populated region of Taiwan were stabilized using our proposed method, with no more large differences in numerical values, whereas the rates in other populous regions were not over-smoothed. Additionally, the stabilized kriging map had improved resolution and helped locate several hot and cold spots in the incidence rates of oral cancer.

**Conclusion:**

We recommend the use of the stabilized kriging method for mapping disease rates.

## INTRODUCTION

Mapping of disease rates is crucial in epidemiological research, and it also helps inform public health policy.^1^ Disease maps are often drawn according to local administrative areas (LAAs), such as counties, cities, or towns. Because different LAAs have different populations, the variances of the disease rate estimates also differ among them (the variances are inversely proportional to population size). Because of larger variances, disease rates are unstable in LAAs with small populations and are prone to appear extremely high or low. Disease maps, therefore, often show large jumps in values, even in adjacent LAAs. The empirical Bayes methods consider differences in variance among different LAAs.^2^^–^^5^ In LAAs with larger variances, disease rates are moved toward the mean to a greater extent, whereas in LAAs with smaller variances, such “shrinkage” is relatively minor. The disease rates can be stabilized and less rugged maps can be drawn using the empirical Bayes methods.

Kriging, a method of spatial interpolation using prior covariances, is often used for mapping environmental data, such as air pollution levels and heavy metal contamination levels in soils.^6^^–^^8^ Krigings can be used to predict values for each coordinate point that is not measured. Therefore, these methods break the constraints of geopolitical boundaries and produce a smooth curved surface in the form of contour lines. However, the kriging methods assume a variance stationary process (the homoscedasticity assumption). This is sensible for mapping air pollution levels or heavy metal contamination levels in soils because it is often the case that each air pollution monitoring station or soil exploration point has the same measurement error or variance. But the assumption is untenable for mapping LAA-based disease rates where the variances are inversely proportional to the population sizes. When “kriging” the disease rates irrespective of the assumption, the resulting rate of the centroid (the geometric center) of an LAA is the original rate in that LAA, but without a stabilizing effect as with the empirical Bayes methods.

The present study proposes a stabilized kriging method to map disease rates. This modified method allows different errors in different LAAs, permitting the rates of the centroids of the LAAs to be appropriately shrunk to the mean and thereby stabilized. Additionally, this method retains the advantages of traditional krigings (ie, breaking geopolitical boundary constraints and producing smooth, curved contour lines). The method is easy to implement. We perform Monte Carlo simulations to compare the statistical properties of the four types of methods (original LAA-based method, empirical Bayes methods, traditional krigings, and our stabilized kriging method). For a demonstration, we used it to draw a map of oral cancer incidence rates in men in Taiwan.

## METHODS

### Description and derivation of the stabilized kriging method

Let the case number for a certain disease and the population size be *d_i_* and *p_i_*, respectively, in the *i*th LAA ( i=1,2,…,n ). The estimated rate is then ${\hat{r}}_{i}=\frac{d_{i}}{p_{i}}$ , and the estimated logarithmic rate, ${\hat{\theta }}_{i}=\log({\hat{r}}_{i})$ , for i=1,2,…,n . In the sequel, we assume the following:

1. The true values of the logarithmic rates *θ_i_*’s follow a random-effects model,^9^^,^^10^ with a mean of *μ* and a variance of *τ*^2^.
2. The covariance between *θ_i_* and *θ_j_* (*i* ≠ *j*) depends only on the distance *D_ij_* between the centroids of the two LAAs, irrespectively of the spatial orientation.
3. The covariance is monotonically decreasing (to zero) as the distance increases (to infinity).
4. The estimated logarithmic rate of the *i*th LAA is its true value plus error: ${\hat{\theta }}_{i}=\theta_{i}+\varepsilon_{i}$ .
5. *E*(*ε_i_*) = 0 and *Cov*(*ε_i_*, *ε_j_*) = 0(*i* ≠ *j*), that is, the estimates of the logarithmic rates are unbiased and are independent of one another.
6. The occurrence of the disease follows a Poisson distribution. Therefore, *Var*(*d_i_*) = *E*(*d_i_*) ≈ *d_i_*, where the observed case numbers, *d_i_*’s, are plugged in to be the expected case numbers, and asymptotically, $v_{i}=\text{Var}(\varepsilon_{i})=\text{Var}[\log({\hat{r}}_{i})]\approx \frac{\text{Var}({\hat{r}}_{i})}{{\hat{r}}_{i}^{2}}=\frac{E(d_{i})}{{\hat{r}}_{i}^{2}p_{i}^{2}}\approx \frac{1}{d_{i}}$ , where the population sizes, *p_i_*’s, are assumed to be error-free.

Note that the traditional kriging methods also invoke the above assumptions 1 to 5.

Subsequently, we use the data of all *n* LAAs ( ${\hat{\theta }}_{i}$ and *v**_i_*, i=1,2,…,n ) to estimate the disease rate in a certain locality (subscripted “0”; this can be one of the LAA centroids or any noncentroid point). We consider a weighted average-type estimator: ${\hat{\theta }}_{0}=\sum_{i=1}^{n}w_{i}{\hat{\theta }}_{i}$ , where $\sum_{i=1}^{n}w_{i}=1$ (the estimated rate in this locality is ${\hat{r}}_{0}=\exp({\hat{\theta }}_{0})$ ). Under the condition of minimum variance unbiased estimation, we can use the Lagrange multiplier method^11^ to determine the weights *w**_i_*’s (*λ* is the Lagrange multiplier, the purpose of which is to guarantee $\sum_{i=1}^{n}w_{i}=1$ ) as follows (see eMaterials 1 for the derivation):

$$\left[\begin{array}{c} w_{1}\\ w_{2}\\ \vdots \\ w_{n}\\ \lambda \end{array}\right]={\left[\begin{array}{ccccc} \tau^{2}+v_{1} & C_{12} & \cdots & C_{1n} & 1\\ C_{21} & \tau^{2}+v_{2} & \cdots & C_{2n} & 1\\ \vdots & \vdots & \ddots & \vdots & \vdots \\ C_{n1} & C_{n2} & \cdots & \tau^{2}+v_{n} & 1\\ 1 & 1 & \cdots & 1 & 0 \end{array}\right]}^{-1}\left[\begin{array}{c} C_{10}\\ C_{20}\\ \vdots \\ C_{n0}\\ 1 \end{array}\right].$$ (1)

The unknown parameters *τ*^2^, *C_ij_*, and *C_i_*_0_ (*C_ji_* = *C_ij_*; i=1,…,n , j=i+1,…,n ) in (1) can be estimated from a semivariogram. The abscissa of the semivariogram is the distance between the centroids of two LAAs, and the ordinate is the semivariance. For *n* LAAs, there are a total of $\frac{n(n-1)}{2}$ points on the semivariogram with coordinates of (*D_ij_*, $\frac{({\hat{\theta }}_{i}-{\hat{\theta }}_{j}{)}^{2}}{2}$ ), for i=1,…,n and j=i+1,…,n . First, we fit a monotonically increasing semivariance function to these points. Commonly used semivariance functions include the spherical function, the circular function, and the exponential function,^6^^–^^8^ which approach a maximum, the “*sill*” as is often called when the distance between LAAs becomes very large. In this study, we used the spherical function for demonstration. Note that the principle is entirely the same for all semivariance functions chosen. The so-called “empirical Bayesian kriging”^12^ automates the estimation of the semivariance function. This can also be used here.

Next, we derive the covariance function as the *sill* minus the semivariance function per se. The covariance function is then a monotonically decreasing function of distance (for the above assumption 3). The value of the covariance function when the distance is 0 is the estimated value of *τ*^2^. Substituting *D_ij_* and *D_i_*_0_ into the covariance function, we obtain *C_ij_* and *C_i_*_0_ (*C_ji_* = *C_ij_*; i=1,…,n , j=i+1,…,n ).

Compared with the traditional krigings, the stabilized kriging method proposed here allows for different variances in different LAAs, the $v_{1},\ldots ,v_{n}$ terms in the diagonal of the square matrix in formula (1). Note that if the case number in a particular LAA (say, the *k*th LAA) is zero (*d_k_* = 0), the estimated logarithmic rate for that LAA will approach negative infinity ( ${\hat{\theta }}_{k}=\log(\frac{d_{k}}{p_{k}})\to -\infty$ ). This will pose a problem for the traditional krigings but not for the stabilized kriging. From formula (1), we see that as the variance in a particular *k*th LAA approaches infinity ( $v_{k}=\frac{1}{d_{k}}\to \infty$ ), the weight apportioned to the LAA will be automatically set to zero (*w**_k_* = 0).

The stabilized kriging method is easy to implement through the following steps:

I. Produce a semivariogram and derive the semivariance function. The same software package for traditional krigings (such as in ^12^) can be used here for this purpose.
II. Derive the covariance function. This is simply the *sill* (the maximum of the semivariance function) minus the semivariance function per se.
III. Obtain the parameters, τ^2^, *C_ij_*, and *C_i_*_0_ (*C_ji_* = *C_ij_*; i=1,…,n , j=i+1,…,n ) from the covariance function. This is a simple readout of the values from the covariance function.
IV. Calculate the kriging weights for any locality to be “kriged”. This can be done using any statistical package that supports matrix calculations for formula (1).
V. Obtain the kriging rates. This is a simple weighted average followed by exponentiation.

### Simulation study

**Figure 1.**
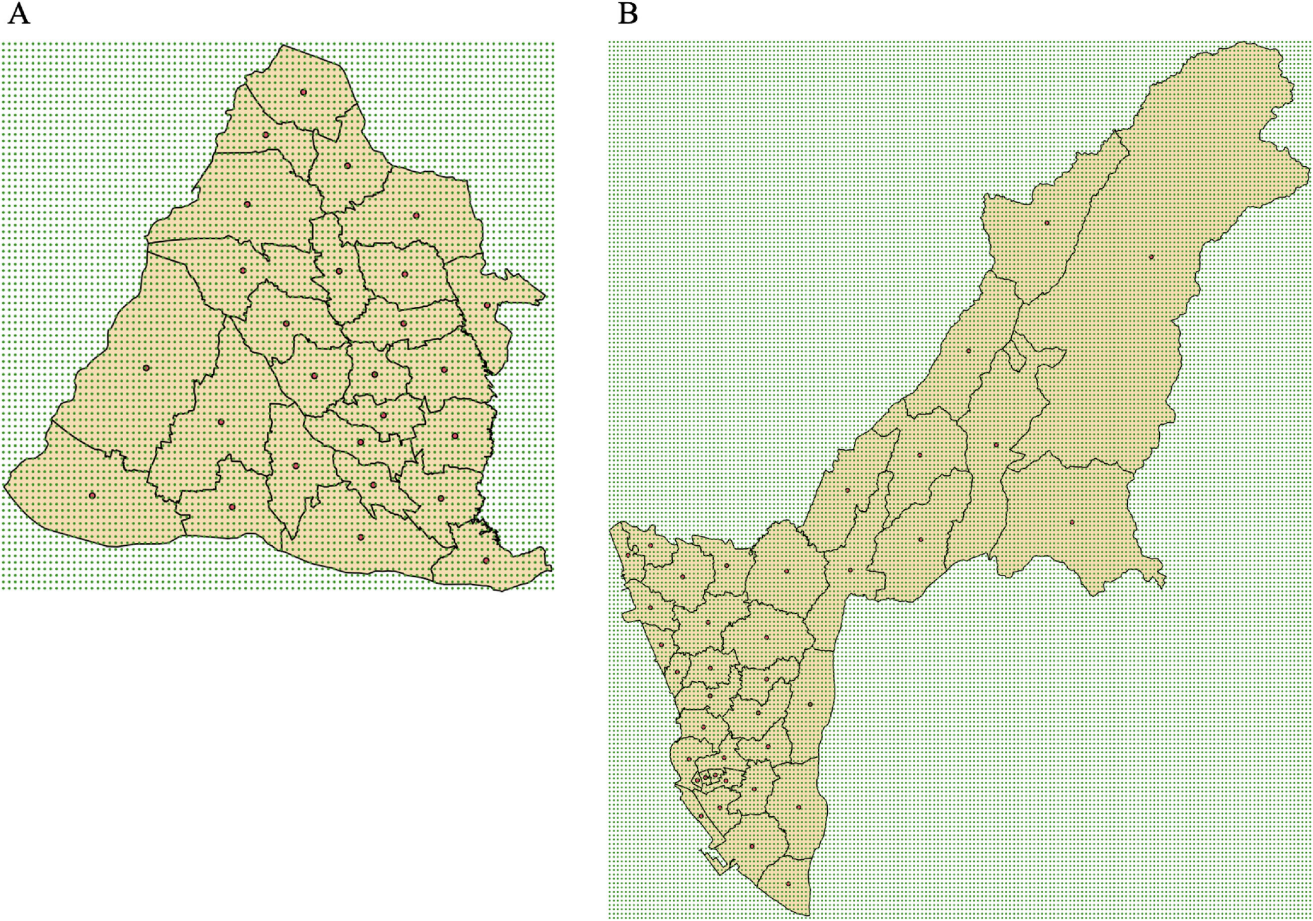
Two counties (A: Changhua County and its 26 local administrative areas; B: Kaohsiung City and County and its 38 local administrative areas) used in the simulation (green dots: coordinate points; red dots: centroids).

We used the geopolitical boundaries of Changhua County (in the midwestern part of Taiwan) and its 26 LAAs (Figure 1A) for simulation. We generated a square mesh coordinate point system (7990 coordinate points with longitudes between 120.2° and 120.7°, and latitudes between 23.79° and 24.21°) to encompass the entire Changhua County (Figure 1A). We randomly selected one of these points and set the true value of the logarithmic rate at that locality to −6.725 (the true value of the rate is *e*^−6.725^ = 120 × 10^−5^). We used a two-dimensional Gaussian function to gradually reduce the true value of the logarithmic rate with distance from the maximum value of −6.725 at this point to a minimum value of −7.725 (*e*^−7.725^ = 44.16 × 10^−5^) (eMaterials 2), representing a single hotspot with the disease rate slowly decreasing outward. We also simulated the scenario of a single hotspot with the disease rate rapidly decreasing outward (eMaterials 2). Besides, we simulated the scenario of double hotspots, where the positions of the two hotspots were random and each one was assigned a rapidly decaying Gaussian function; the logarithmic rate’s true value was the average of the two (see eMaterials 2).

We used a log-normal distribution to generate the population size of every LAA in Changhua County. We considered scenarios where the expected values of the population size were large (100,000), moderate (50,000), and small (25,000); the coefficients of variation of the population size were small (0.1), moderate (0.5), and large (1.0), for 3 × 3 = 9 scenarios.

For an LAA, we multiplied its (simulated) population size by the (simulated) disease rate at the centroid of that LAA (Figure 1A) to obtain the expected case number. We then used a Poisson distribution to generate the observed case number of that LAA. The estimated logarithmic rate of the LAA is the logarithm of the ratio between the observed case number and the population size.

We used the stabilized kriging method to estimate the rates of all coordinate points within the geopolitical boundary of Changhua County (a total of 4,417 coordinate points) based on the simulated data of 26 LAAs ( ${\hat{\theta }}_{i}$ and *v**_i_*, i=1,…,26 ). For comparison, we performed traditional krigings with and without adjusting for the nugget effect. In nugget-adjusted kriging, the “*nugget*”, the value of the semivariance function when the distance is 0, was added to the diagonal of the square matrix in formula (1) to allow for measurement errors of the 26 LAAs. Furthermore, we used the original rate and the empirical Bayes-stabilized rate of an LAA to estimate the rates of all coordinate points within that LAA (ie, all coordinate points in the same LAA had the same rate). Four different approaches were used to generate the empirical Bayes estimates. In the first approach, no assumption was made for the prior distribution of the log disease rates, and the prior mean and variance were estimated using the DerSimonian-Laird method.^9^ In the second approach, a Poisson-gamma model (a gamma prior and a Poisson error)^2^^–^^5^ was assumed and the scale and shape parameters of the gamma distribution were estimated using the maximum likelihood method. The third and the fourth approaches were similar to the first and the second, respectively, except that the hyperparameters of an LAA were estimated locally from the data of its 15 nearest LAAs and itself.

We quantified the error of each method with the index of symmetric mean absolute percentage error (SMAPE)^13^^,^^14^: $\text{SMAPE}=\frac{100\%}{4417}\sum_{j=1}^{4417}\frac{|{\hat{r}}_{j}-r_{j}|}{{\hat{r}}_{j}+r_{j}}$ . The smaller the SMAPE index, the closer the estimated value is to the true value (mean squared error [MSE] is also a commonly used error metric; however, the same degree of error as quantified by MSE has different implications in high-rate vs low-rate areas). We performed 1,000 simulations for each scenario.

Simulations were also performed for another city/county with a different geometric shape, the Kaohsiung City and County (Figure 1B; 35,552 coordinate points with longitudes between 120.17° and 121.05°, and latitudes between 22.47° and 23.47°; 10,564 coordinate points within the city/county; a total 38 LAAs).

### Real-world data analysis

We used oral cancer incidence in men in Taiwan as an example to demonstrate the methodology. All male patients with incident oral cancer (ICD-O-3: C00–C06, C09–C10, C12–C14) from 2012 to 2016 were extracted from the Taiwan Cancer Registry. The registry is a nationwide population-based cancer registry system in Taiwan with high data quality and completeness.^15^^,^^16^ For privacy protection, the registry collects the information of the LAA a cancer patient resides in but not his/her exact address. The population numbers for men in every LAA in Taiwan from 2012 to 2016 were extracted from an online database provided by the Department of Statistics of the Ministry of the Interior in Taiwan. As household registration in Taiwan is mandatory, the population numbers can be regarded as exact.

The World Health Organization’s 2000 World Standard Population proportions were used as the standard population to calculate the directly age-standardized incidence rate (DASIR; eMaterials 3 presents the formulas of DASIR and the variance of logDASIR). We restricted our analysis to the 349 LAAs in the main island of Taiwan (19 LAAs in offshore islands being excluded).

First, we used the DASIRs of the 349 LAAs to produce a map. Next, we used the empirical Bayes methods to stabilize the maps. The DASIRs, being weighted sums (rather than simple sums) of Poisson rate parameters, are not Poisson distributed by themselves.^17^ We, therefore, considered only the empirical Bayes methods that do not assume distribution forms (the first and the third approaches described in the previous section). Finally, we used the traditional krigings (with and without adjusting for the nugget effect) and our proposed stabilized kriging to produce the maps.

## RESULTS

### Simulation results

The simulation results using the geopolitical boundaries of Changhua County and its 26 LAAs are presented in Table 1, Table 2, and Table 3. Stabilized kriging was superior to (having smaller SMAPE than) all other methods in all scenarios considered, except for the double-hotspots scenario when the expected population size was small, and the coefficient of variation, large (the last row of Table 3, where the “local” Poisson-gamma empirical Bayes method had the smallest SMAPE of 6.42%, and the stabilized kriging, the second smallest SMAPE of 6.55%).

**Table 1.** The symmetric mean absolute percentage errors (%) of the various methods in the scenario of a single hotspot with the disease rate slowly decreasing outward for the geopolitical boundaries of Changhua County and its 26 local administrative areas

Population Size		Methods							

Mean	Coefficient of variation	Original rate	Empirical Bayes - 1^a^	Empirical Bayes - 2^b^	Empirical Bayes - 3^c^	Empirical Bayes - 4^d^	Traditional kriging - 1^e^	Traditional kriging - 2^f^	Stabilized kriging
100,000	0.1	5.27	4.85	4.86	4.66	4.67	3.48	3.20	2.90
100,000	0.5	5.66	5.06	5.07	4.82	4.82	3.88	3.54	3.08
100,000	1.0	6.65	5.54	5.54	5.15	5.15	4.92	4.42	3.59
50,000	0.1	7.02	6.01	6.02	5.63	5.61	5.12	4.51	4.09
50,000	0.5	7.62	6.24	6.23	5.76	5.73	5.66	4.93	4.29
50,000	1.0	9.05	6.66	6.62	6.01	5.94	7.08	6.28	5.11
25,000	0.1	9.75	7.51	7.45	6.73	6.60	7.45	6.41	5.82
25,000	0.5	10.59	7.68	7.58	6.83	6.70	8.24	7.19	6.23
25,000	1.0	12.82	8.12	7.97	7.10	6.91	10.08	8.81	6.63

^a^No assumption for the prior distribution; prior mean and variance estimated from all 26 local administrative areas.

^b^Poisson-gamma model; scale and shape parameters of the gamma distribution estimated from all 26 local administrative areas.

^c^No assumption for the prior distribution; prior mean and variance for a local administrative area estimated from the data of its 15 nearest local administrative areas and itself.

^d^Poisson-gamma model; scale and shape parameters of the gamma distribution for a local administrative area estimated from the data of its 15 nearest local administrative areas and itself.

^e^Without adjusting for the nugget effect.

^f^Adjusted for the nugget effect.

**Table 2.** The symmetric mean absolute percentage errors (%) of the various methods in the scenario of a single hotspot with the disease rate rapidly decreasing outward for the geopolitical boundaries of Changhua County and its 26 local administrative areas

Population Size		Methods							

Mean	Coefficient of variation	Original rate	Empirical Bayes - 1^a^	Empirical Bayes - 2^b^	Empirical Bayes - 3^c^	Empirical Bayes - 4^d^	Traditional kriging - 1^e^	Traditional kriging - 2^f^	Stabilized kriging
100,000	0.1	6.45	5.35	5.39	4.93	4.92	4.20	3.96	3.59
100,000	0.5	6.89	5.54	5.60	5.12	5.11	4.59	4.27	3.76
100,000	1.0	8.01	5.83	5.93	5.36	5.36	5.63	5.17	4.20
50,000	0.1	8.60	6.38	6.50	5.98	5.97	5.90	5.37	4.78
50,000	0.5	9.13	6.51	6.62	6.03	6.01	6.38	5.74	4.99
50,000	1.0	11.00	7.01	7.10	6.44	6.39	8.07	7.25	5.69
25,000	0.1	11.69	7.63	7.69	7.14	7.03	8.19	7.15	6.33
25,000	0.5	12.69	7.82	7.82	7.27	7.10	8.92	7.83	6.52
25,000	1.0	15.44	8.43	8.20	7.72	7.39	11.53	10.02	7.37

^a^No assumption for the prior distribution; prior mean and variance estimated from all 26 local administrative areas.

^b^Poisson-gamma model; scale and shape parameters of the gamma distribution estimated from all 26 local administrative areas.

^c^No assumption for the prior distribution; prior mean and variance for a local administrative area estimated from the data of its 15 nearest local administrative areas and itself.

^d^Poisson-gamma model; scale and shape parameters of the gamma distribution for a local administrative area estimated from the data of its 15 nearest local administrative areas and itself.

^e^Without adjusting for the nugget effect.

^f^Adjusted for the nugget effect.

**Table 3.** The symmetric mean absolute percentage errors (%) of the various methods in the scenario of double hotspots for the geopolitical boundaries of Changhua County and its 26 local administrative areas

Population Size		Methods							

Mean	Coefficient of variation	Original rate	Empirical Bayes - 1^a^	Empirical Bayes - 2^b^	Empirical Bayes - 3^c^	Empirical Bayes - 4^d^	Traditional kriging - 1^e^	Traditional kriging - 2^f^	Stabilized kriging
100,000	0.1	5.95	4.76	4.78	4.59	4.48	4.15	3.80	3.45
100,000	0.5	6.38	4.90	4.93	4.61	4.61	4.52	4.13	3.63
100,000	1.0	7.62	5.16	5.18	4.76	4.75	5.58	5.00	4.07
50,000	0.1	8.19	5.72	5.72	5.37	5.32	5.85	5.08	4.60
50,000	0.5	8.92	5.83	5.83	5.44	5.38	6.41	5.59	4.86
50,000	1.0	10.52	6.21	6.14	5.69	5.56	7.81	6.89	5.43
25,000	0.1	11.34	6.84	6.69	6.38	6.20	7.86	6.77	6.03
25,000	0.5	12.34	6.92	6.74	6.44	6.25	8.66	7.35	6.18
25,000	1.0	15.36	7.29	7.00	6.72	6.42	11.01	9.50	6.55

^a^No assumption for the prior distribution; prior mean and variance estimated from all 26 local administrative areas.

^b^Poisson-gamma model; scale and shape parameters of the gamma distribution estimated from all 26 local administrative areas.

^c^No assumption for the prior distribution; prior mean and variance for a local administrative area estimated from the data of its 15 nearest local administrative areas and itself.

^d^Poisson-gamma model; scale and shape parameters of the gamma distribution for a local administrative area estimated from the data of its 15 nearest local administrative areas and itself.

^e^Without adjusting for the nugget effect.

^f^Adjusted for the nugget effect.

The SMAPEs of the original rate and traditional krigings increased with decreasing population size and increasing coefficient of variation. By comparison, the performance of the stabilized kriging and empirical Bayes methods was fairly robust to population sizes and variations. With larger population sizes and smaller variations, traditional krigings outperformed empirical Bayes methods. With smaller population sizes and larger variations, the converse was true.

The simulation results using the geopolitical boundaries of Kaohsiung City and County and its 38 LAAs are presented in eTable 1, eTable 2, and eTable 3. The findings are similar to those of Changhua County and its 26 LAAs (Table 1, Table 2, and Table 3).

### Oral cancer incidence in men in Taiwan

In the 349 LAAs in the main island of Taiwan, the total number of cases (men diagnosed with incident oral cancer from 2012 to 2016) was 34,619 (99.2 cases per LAA; min = 1; max = 699), and the total population number (men; 5-year average between 2012 and 2016) was 11,558,438 (33,118.7 population per LAA; min = 933; max = 272,602; geographical distribution: shown in eFigure 1).

**Figure 2.**
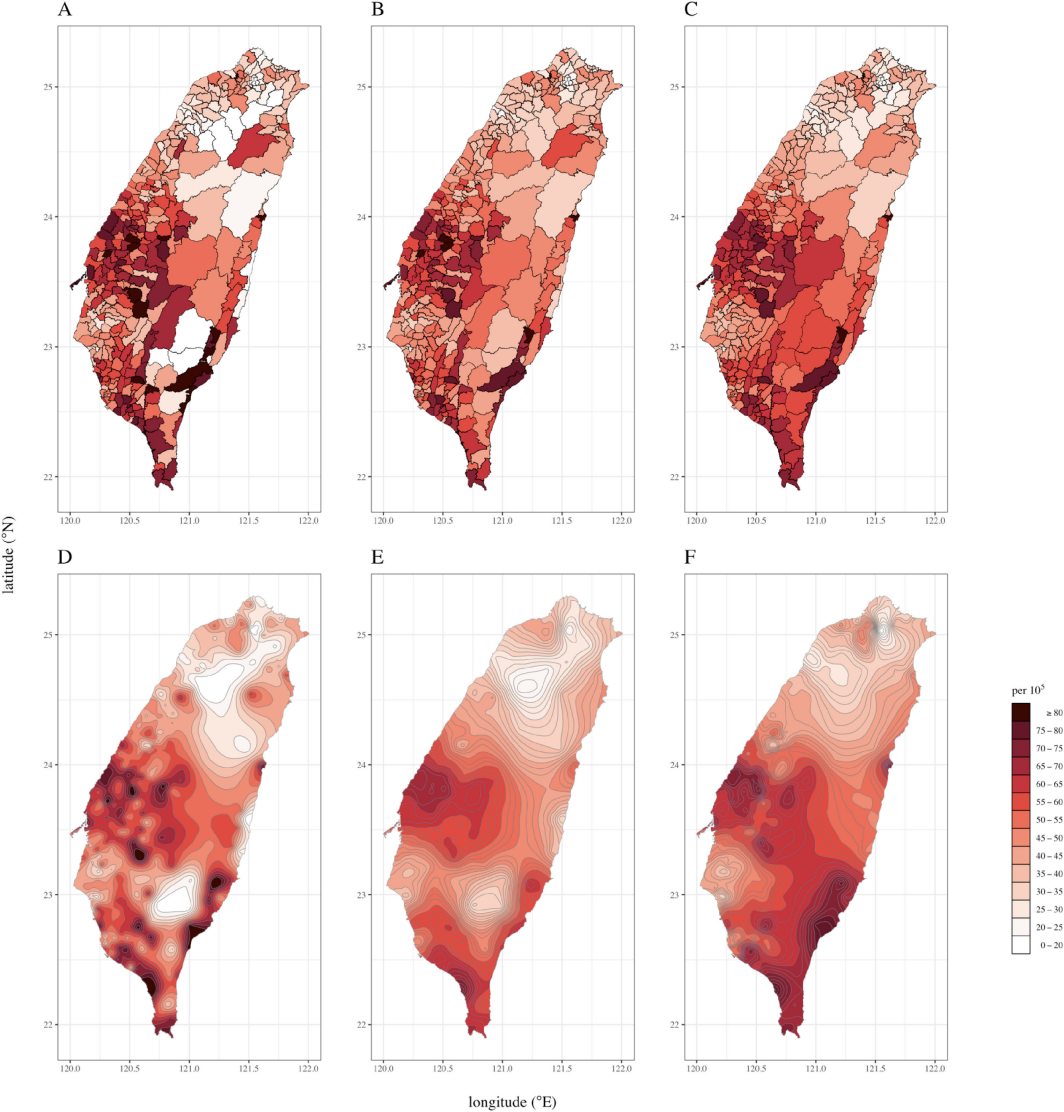
Maps of the age-standardized incidence rate of oral cancer in men in Taiwan; only the data of the 349 local administrative areas in the main island of Taiwan being analyzed and shown (A: original rate; B: empirical Bayes method with the prior mean and variance estimated from all local administrative areas; C: empirical Bayes method with the prior mean and variance for a local administrative area estimated from the data of its 15 nearest local administrative areas and itself; D: traditional kriging without adjusting for the nugget effect; E: traditional kriging adjusted for the nugget effect; F: stabilized kriging method).

The original DASIR map (Figure 2A) reveals that the areas with high rates are mostly distributed in the central, southern, and southeastern regions. A comparison of the map produced by the empirical Bayes methods (Figure 2B and Figure 2C) and the original LAA-based map (Figure 2A) reveals that the rates of those LAAs with small population sizes and extremely high or low values of rates (in the central mountainous region) were shrunk toward the average value and hence were stabilized.

Figure 2D demonstrates that while the traditional kriging without adjusting for the nugget effect produces a smooth curved surface in the form of contour lines, kriging rates at the centroids of the LAAs were not stabilized; the extremely high or low rates in the central mountainous sparsely-populated region remained unchanged. The nugget-adjusted kriging does stabilize the rates, but indiscriminately; the rates in densely and sparsely populated LAAs are being stabilized to the same extent. Consequently, the method produces an over-smoothed map (Figure 2E). Figure 2F is the map produced using the stabilized kriging method, which appropriately shrinks the rates of those LAAs with small population sizes and extreme values of rates to the average value. Compared with the traditional krigings (Figure 2D and Figure 2E), the rates in the central mountainous sparsely-populated region of Taiwan are stabilized, with no more large differences in numerical values, whereas the rates in other populous regions are not over smoothed.

With the confusing signals in the central mountainous sparsely-populated region of Taiwan removed, the stabilized kriging map (Figure 2F) reveals that the age-standardized incidence rates of oral cancer in men in central and southern Taiwan are higher than those in other parts of Taiwan. The stabilized kriging method also breaks the constraints of geopolitical boundaries and achieves better resolution. We can discern hot spots in the mid-western region and the southern, southeastern, and mid-eastern coastal areas of Taiwan, as well as the cold spot in the southwestern plain.

In the stabilized kriging analysis (Figure 2F), the representative points of LAAs were taken to be their respective geometric centers. eFigure 2 presents the results when the “population centroids” (the mean centers of the population) were representing the LAAs, and eFigure 3 presents the results when the geometric centers of the two most populated regions (boroughs or villages) within an LAA were jointly representing the LAA. The findings are essentially the same as those in Figure 2F. These are as expected since the median distance between the geometric and population centers of the LAAs in Taiwan is only 1.1 km. There are some LAAs where the two centers are farther apart (maximally 11.7 km apart). But these are the LAAs in the central mountainous sparsely-populated region of Taiwan (eFigure 4) where the rates would be stabilized using our proposed method anyway. If this is not the case, one may need to let more population subcenters jointly represent an LAA or better still, obtain the disease rates in the subcenters themselves if possible.

## DISCUSSION

In epidemiology, the production of accurate disease maps is important, as such data can help inform choices for public health policy. Krigings, which are often used to map environmental data and estimate missing data points, make assumptions that often fail for LAA-based data, such as local disease rates, as the variances may differ substantially between localities. The empirical Bayes methods properly account for differences in variance between LAAs and stabilize rate estimates, but the methods lack the powerful vantage of krigings (ie, breaking geopolitical boundary constraints and producing smooth, curved contour lines). In this paper, we proposed an easy-to-implement stabilized kriging method that combines the advantages of traditional krigings and the empirical Bayes methods. We compared our proposed methods to two variants of boundary-breaking traditional krigings and four variants of boundary-respecting empirical Bayes methods. The “SpatialEpi” functionality in the R package (R Foundation for Statistical Computing, Vienna, Austria) implemented two other boundary-respecting methods: the conditional autoregressive (CAR) and simultaneous autoregressive (SAR) models. Both models take the spatial correlations between LAAs (or the spatial configurations of the LAAs) into account.^5^ The local empirical Bayes methods, the third and the fourth approaches in this paper, also address the spatial correlation/configuration issues, though not as sophisticatedly as do the CAR and SAR models.

As described in this paper, the stabilized kriging method only requires point estimates ( ${\hat{\theta }}_{i}$ ’s) and the corresponding variances (*v**_i_*’s), regardless of what these represent. In addition to mapping disease rates, the stabilized kriging method can also map other factors, such as average income, smoking prevalence, gene frequency, and blood type distributions. However, both the stabilized and traditional kriging methods have limitations, such as when the disease rates are constrained by natural barriers (eg, high mountains or wide rivers) or are driven by aggregate-level factors and follow geopolitical boundaries (eg, when different counties/cities have different levels of medical care). To address the issue, one can set the distance between two points on opposite sides of a barrier or a boundary to be arbitrarily large or consider alternative distance metrics, such as travel time or travel costs.

The stabilized kriging method may produce over-smoothed results for regions with very small population sizes. For example, in Figure 2F the rates in the central and southern mountain regions were shrunk to the average value, and it is difficult to determine where (or even whether) there is any hot or cold spot there. Conversely, over-smoothing is negligible in populous regions such as the northern, midwestern, and southwestern metropolitan areas and the eastern coastal towns. The shrinkage resulting from stabilized kriging scales inversely with population size. The matrix formula of the stabilized kriging method indicates that when the population size of an LAA is very large (the variance is nearly zero), the estimated value of the stabilized kriging at the centroid of that LAA approaches the value obtained using the traditional kriging without adjusting for the nugget effect (ie, there is no shrinkage). Trend surface analysis, which uses a polynomial surface to fit the data,^18^ is also commonly used to map disease rates. However, this method is prone to produce edge effects^19^ (where extrapolated values at the edges of the map are extremely high or low), which confounds reading the map. For the stabilized kriging method, the predicted rate at a locality is the weighted average of the rates of the nearby LAAs (the closer an LAA is to that locality, the greater the weight is); because this also holds at the edges of the map, our method avoids the edge effect problem.

In our case study, the stabilized kriging map (Figure 2F) revealed that the age-standardized oral cancer incidence in men in central and southern Taiwan is higher than that in other parts of Taiwan, with hot spots in the mid-western region and the southern, southeastern, and mid-eastern coastal areas. This may be related to the high prevalence of betel nut chewing in the aforementioned areas, while the cold spot in the southwest plain may be explained by the relative infrequence of betel nut chewing in that area (eFigure 5). The high concentrations of heavy metals in the midwestern region’s soils^20^ may also contribute to the hot spots located there. Here, the stabilized map combines the advantage of traditional krigings and the empirical Bayes methods. Further studies are needed to extend the methodology to stabilize the map produced from the so-called “universal krigings”,^6^^–^^8^ which can incorporate covariate information, such as betel nut chewing and heavy metal concentrations.

In conclusion, the stabilized kriging method introduced in this study is easy to implement. We demonstrated through Monte Carlo simulation that the method has desirable statistical performance. The method also helped identify several hot and cold spots in a case study of the incidence rates of oral cancer in men in Taiwan. Based on these results, we recommend using the stabilized kriging method for mapping not only disease rates but any types of quantitatively similar data that meet the method’s assumptions.

## SUPPLEMENTARY MATERIAL

eMaterials 1. Derivation of the stabilized kriging formula

eMaterials 2. Simulation scenarios

eMaterials 3. Formulas of the directly age-standardized incidence rate (DASIR) and the variance of logDASIR

eTable 1. The symmetric mean absolute percentage errors (%) of the various methods in the scenario of a single hotspot with the disease rate slowly decreasing outward for the geopolitical boundaries of Kaohsiung City and County and its 38 local administrative areas

eTable 2. The symmetric mean absolute percentage errors (%) of the various methods in the scenario of a single hotspot with the disease rate rapidly decreasing outward for the geopolitical boundaries of Kaohsiung City and County and its 38 local administrative areas

eTable 3. The symmetric mean absolute percentage errors (%) of the various methods in the scenario of double hotspots for the geopolitical boundaries of Kaohsiung City and County and its 38 local administrative areas

eFigure 1. Population numbers (men; five-year average between 2012 and 2016) in the 349 local administrative areas in the main island of Taiwan

eFigure 2. Stabilized kriging analysis when the population centroids were representing the local administrative areas

eFigure 3. Stabilized kriging analysis when the geometric centers of the two most populated regions (boroughs or villages) within a local administrative area were jointly representing the local administrative area

eFigure 4. Distances between the geometric and population centers of the 349 local administrative areas in the main island of Taiwan

eFigure 5. Prevalence of betel nuts chewing in various counties and cities in the main island of Taiwan in 2017
