# Supplementary material for: A Stabilized Kriging Method for Mapping Disease Rates
Source: J Epidemiol. 2023 Apr 5;33(4):201–8. doi: 10.2188/jea.JE20210276 (PMC9939925; doi:10.2188/jea.JE20210276)

eMaterials 1. Derivation of the stabilized kriging formula

The mean square error of the estimated value is $E(\hat{\theta}_0 - \theta_0)^2$, and the Lagrange function is as follows:

$$\mathrm{L}(w_1, w_2, \dots, w_n, \lambda) = E(\hat{\theta}_0 - \theta_0)^2 + 2\lambda(\textstyle\sum_{i=1}^{n} w_i - 1)$$

$$= Var(\hat{\theta}_0 - \theta_0) + \left[E(\hat{\theta}_0 - \theta_0)\right]^2 + 2\lambda(\textstyle\sum_{i=1}^{n} w_i - 1)$$

$$= Var\left(\textstyle\sum_{i=1}^{n} w_i\,(\theta_i + \varepsilon_i)\right) - 2Cov(\textstyle\sum_{i=1}^{n} w_i(\theta_i + \varepsilon_i), \theta_0) + Var(\theta_0) + 0 + 2\lambda(\textstyle\sum_{i=1}^{n} w_i - 1)$$

$$= Var(\textstyle\sum_{i=1}^{n} w_i\,\theta_i) + Var(\textstyle\sum_{i=1}^{n} w_i\,\varepsilon_i) - 2\textstyle\sum_{i=1}^{n} w_i\,Cov(\theta_i, \theta_0) + \tau^2 + 2\lambda(\textstyle\sum_{i=1}^{n} w_i - 1)$$

$$= (\textstyle\sum_{i=1}^{n} w_i{}^2)\tau^2 + \textstyle\sum_{i=1}^{n}\sum_{j\neq i} w_i w_j\,C_{ij} + \textstyle\sum_{i=1}^{n} w_i{}^2\,v_i - 2\textstyle\sum_{i=1}^{n} w_i\,C_{i0} + \tau^2 + 2\lambda(\textstyle\sum_{i=1}^{n} w_i - 1).$$

The first-order partial derivatives of this function with respect to w_i (i=1,2,..., n) and λ are set to zero, as follows:

$$\frac{\partial \mathrm{L}(w_1,w_2,\dots,w_n,\lambda)}{\partial w_i} = 2w_i\tau^2 + 2\textstyle\sum_{j\neq i} w_j C_{ij} + 2w_i v_i - 2C_{i0} + 2\lambda = 0, \quad \text{for } i=1,2,\dots,n,$$

and

$$\frac{\partial \mathrm{L}(w_1,w_2,\dots,w_n,\lambda)}{\partial \lambda} = 2(\textstyle\sum_{i=1}^{n} w_i - 1) = 0.$$

These equations can be cast in matrix form, as follows:

$$\begin{bmatrix} \tau^2 & C_{12} & \cdots & C_{1n} & 1 \\ C_{21} & \tau^2 & \cdots & C_{2n} & 1 \\ \vdots & \vdots & \ddots & \vdots & \vdots \\ C_{n1} & C_{n2} & \cdots & \tau^2 & 1 \\ 1 & 1 & \cdots & 1 & 0 \end{bmatrix} \begin{bmatrix} w_1 \\ w_2 \\ \vdots \\ w_n \\ \lambda \end{bmatrix} + \begin{bmatrix} v_1 & 0 & \cdots & 0 & 0 \\ 0 & v_2 & \cdots & 0 & 0 \\ \vdots & \vdots & \ddots & \vdots & \vdots \\ 0 & 0 & \cdots & v_n & 0 \\ 0 & 0 & \cdots & 0 & 0 \end{bmatrix} \begin{bmatrix} w_1 \\ w_2 \\ \vdots \\ w_n \\ \lambda \end{bmatrix} - \begin{bmatrix} C_{10} \\ C_{20} \\ \vdots \\ C_{n0} \\ 1 \end{bmatrix} = \begin{bmatrix} 0 \\ 0 \\ \vdots \\ 0 \\ 0 \end{bmatrix}.$$

From this, we obtain

$$\begin{bmatrix} w_1 \\ w_2 \\ \vdots \\ w_n \\ \lambda \end{bmatrix} = \begin{bmatrix} \tau^2 + v_1 & C_{12} & \cdots & C_{1n} & 1 \\ C_{21} & \tau^2 + v_2 & \cdots & C_{2n} & 1 \\ \vdots & \vdots & \ddots & \vdots & \vdots \\ C_{n1} & C_{n2} & \cdots & \tau^2 + v_n & 1 \\ 1 & 1 & \cdots & 1 & 0 \end{bmatrix}^{-1} \begin{bmatrix} C_{10} \\ C_{20} \\ \vdots \\ C_{n0} \\ 1 \end{bmatrix}.$$

eMaterials 2. Simulation scenarios

Let $D_{a,b}$ denote the distance between two coordinate points a and b (in kilometer). The true values of the logarithmic rate at coordinate point x are listed below for the three scenarios, respectively.

<u>Scenario 1</u> A single hotspot (at coordinate point h) with the disease rate slowly decreasing outward: $\theta_x = -7.725 + \exp\left(-\frac{D_{x,h}^2}{800}\right)$.

<u>Scenario 2</u> A single hotspot (at coordinate point h) with the disease rate rapidly decreasing outward: $\theta_x = -7.725 + \exp\left(-\frac{D_{x,h}^2}{200}\right)$.

<u>Scenario 3</u> Double hotspots (at coordinate point h and h^*, respectively):

$$\theta_x = -7.725 + \frac{1}{2}\exp\left(-\frac{D_{x,h}^2}{200}\right) + \frac{1}{2}\exp\left(-\frac{D_{x,h^*}^2}{200}\right).$$

eMaterials 3. Formulas of the directly age-standardized incidence rate (DASIR) and the variance of logDASIR

Let the age groups be indexed by k ($k = 1, \ldots, K$), and let $w_k > 0$ for $k = 1, \ldots, K$ ($\sum_{k=1}^{K} w_k = 1$) be the weights used in the standardization. Formulas of the directly age-standardized incidence rate (DASIR) and the variance of logDASIR are as below:

$$\text{DASIR} = \sum_{k=1}^{K} w_k \hat{r}_k,$$

and

$$Var(\log\text{DASIR}) \approx \text{DASIR}^{-2} \times \sum_{k=1}^{K} \frac{w_k^2 \hat{r}_k}{p_k},$$

where $\hat{r}_k$ and p_k are the estimated rate and the population size, respectively, in the kth age group.

eTable 1. The symmetric mean absolute percentage errors (%) of the various methods in the scenario of a single hotspot with the disease rate slowly decreasing outward for the geopolitical boundaries of Kaohsiung City and County and its 38 local administrative areas

Population size		Methods							
Mean	Coefficient of variation	Original rate	Empirical Bayes - 1[a]	Empirical Bayes - 2[b]	Empirical Bayes -3[c]	Empirical Bayes - 4[d]	Traditional kriging - 1[e]	Traditional kriging - 2[f]	Stabilized kriging
100,000	0.1	6.79	5.31	5.29	4.96	4.94	4.76	4.47	4.19
100,000	0.5	7.16	5.51	5.51	5.03	5.03	5.21	4.85	4.44
100,000	1.0	8.39	5.88	5.85	5.32	5.29	6.23	5.56	4.73
50,000	0.1	8.75	6.27	6.23	5.79	5.74	6.27	5.52	5.13
50,000	0.5	9.58	6.33	6.27	5.83	5.77	6.86	6.01	5.26
50,000	1.0	11.23	7.12	7.00	6.48	6.38	8.44	7.29	5.91
25,000	0.1	12.08	7.64	7.36	7.01	6.84	8.61	7.30	6.54
25,000	0.5	12.94	8.16	7.93	7.42	7.28	9.85	8.36	6.89
25,000	1.0	15.72	8.48	8.13	7.65	7.40	12.14	10.34	7.33

[a] No assumption for the prior distribution; prior mean and variance estimated from all 38 local administrative areas.
[b] Poisson-gamma model; scale and shape parameters of the gamma distribution estimated from all 38 local administrative areas.
[c] No assumption for the prior distribution; prior mean and variance for a local administrative area estimated from the data of its 15 nearest local administrative areas and itself.
[d] Poisson-gamma model; scale and shape parameters of the gamma distribution for a local administrative area estimated from the data of its 15 nearest local administrative areas and itself.
[e] Without adjusting for the nugget effect.
[f] Adjusted for the nugget effect.

eTable 2. The symmetric mean absolute percentage errors (%) of the various methods in the scenario of a single hotspot with the disease rate rapidly decreasing outward for the geopolitical boundaries of Kaohsiung City and County and its 38 local administrative areas

Population size		Methods							
Mean	Coefficient of variation	Original rate	Empirical Bayes - 1[a]	Empirical Bayes - 2[b]	Empirical Bayes -3[c]	Empirical Bayes - 4[d]	Traditional kriging - 1[e]	Traditional kriging - 2[f]	Stabilized kriging
100,000	0.1	6.90	3.70	3.65	3.81	3.78	4.22	3.49	3.32
100,000	0.5	7.38	3.75	3.71	3.82	3.76	4.62	3.74	3.42
100,000	1.0	8.62	3.84	3.61	3.81	3.76	5.55	4.47	3.58
50,000	0.1	9.05	4.15	4.07	4.43	4.37	5.42	4.24	3.98
50,000	0.5	9.95	4.41	4.33	4.62	4.54	6.16	4.85	4.29
50,000	1.0	11.80	4.53	4.55	4.76	4.63	7.61	6.07	4.50
25,000	0.1	12.72	5.11	4.86	5.43	5.36	7.66	5.77	5.13
25,000	0.5	13.96	5.21	4.88	5.55	5.38	8.76	6.54	5.23
25,000	1.0	16.79	5.40	4.92	5.75	5.62	11.20	8.85	5.58

[a] No assumption for the prior distribution; prior mean and variance estimated from all 38 local administrative areas.
[b] Poisson-gamma model; scale and shape parameters of the gamma distribution estimated from all 38 local administrative areas.
[c] No assumption for the prior distribution; prior mean and variance for a local administrative area estimated from the data of its 15 nearest local administrative areas and itself.
[d] Poisson-gamma model; scale and shape parameters of the gamma distribution for a local administrative area estimated from the data of its 15 nearest local administrative areas and itself.
[e] Without adjusting for the nugget effect.
[f] Adjusted for the nugget effect.

eTable 3. The symmetric mean absolute percentage errors (%) of the various methods in the scenario of double hotspots for the geopolitical boundaries of Kaohsiung City and County and its 38 local administrative areas

Population size		Methods							
Mean	Coefficient of variation	Original rate	Empirical Bayes - 1[a]	Empirical Bayes - 2[b]	Empirical Bayes -3[c]	Empirical Bayes - 4[d]	Traditional kriging - 1[e]	Traditional kriging - 2[f]	Stabilized kriging
100,000	0.1	6.49	3.49	3.48	3.52	3.50	4.15	3.32	3.19
100,000	0.5	6.93	3.51	3.49	3.56	3.51	4.45	3.56	3.28
100,000	1.0	8.24	3.52	3.50	3.59	3.53	5.40	4.25	3.49
50,000	0.1	8.85	4.16	4.03	4.30	4.21	5.47	4.14	3.91
50,000	0.5	9.69	4.16	4.05	4.29	4.22	6.02	4.57	4.04
50,000	1.0	11.57	4.17	4.05	4.31	4.23	7.49	5.69	4.20
25,000	0.1	12.47	4.85	4.49	5.11	4.98	7.40	5.40	4.78
25,000	0.5	13.49	5.01	4.59	5.17	4.99	8.51	6.36	4.98
25,000	1.0	16.99	5.10	4.59	5.38	5.08	11.07	8.48	5.21

[a] No assumption for the prior distribution; prior mean and variance estimated from all 38 local administrative areas.
[b] Poisson-gamma model; scale and shape parameters of the gamma distribution estimated from all 38 local administrative areas.
[c] No assumption for the prior distribution; prior mean and variance for a local administrative area estimated from the data of its 15 nearest local administrative areas and itself.
[d] Poisson-gamma model; scale and shape parameters of the gamma distribution for a local administrative area estimated from the data of its 15 nearest local administrative areas and itself.
[e] Without adjusting for the nugget effect.
[f] Adjusted for the nugget effect.

eFigure 1. Population numbers (men; five-year average between 2012 and 2016) in the 349 local administrative areas in the main island of Taiwan

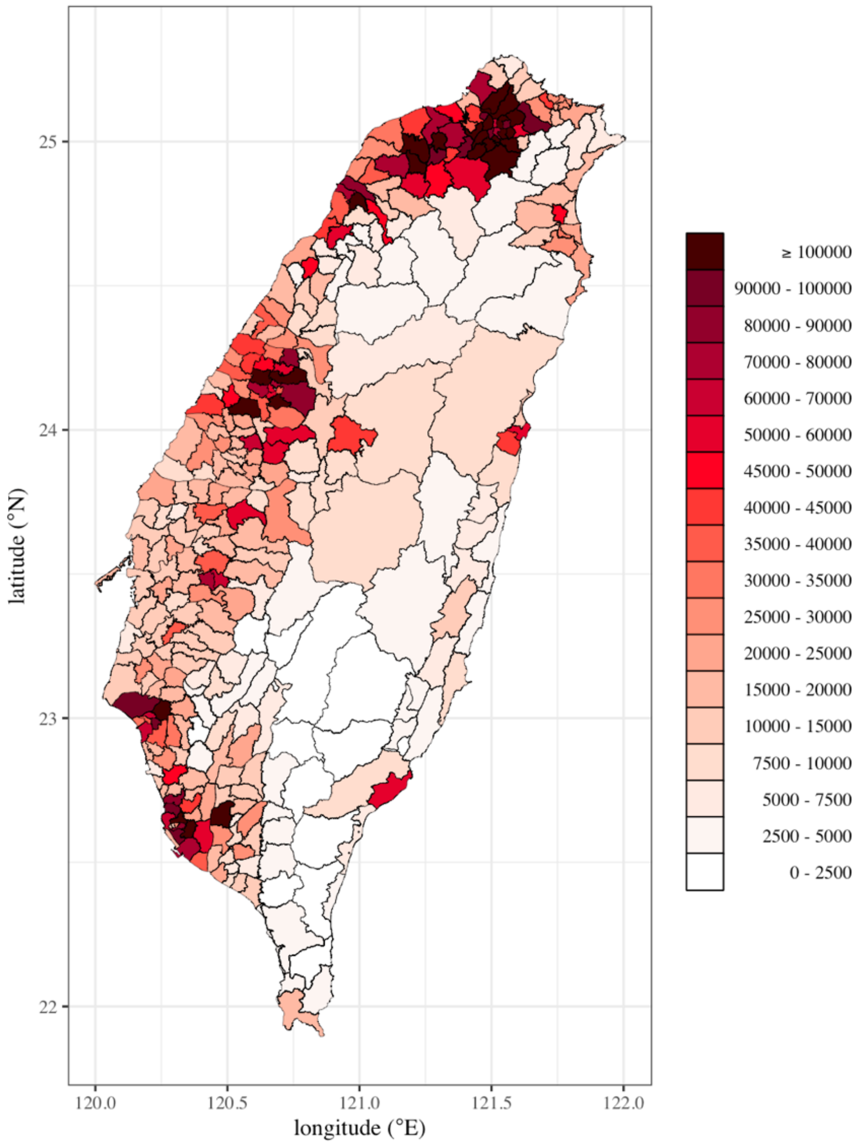

eFigure 2. Stabilized kriging analysis when the population centroids were representing the local administrative areas

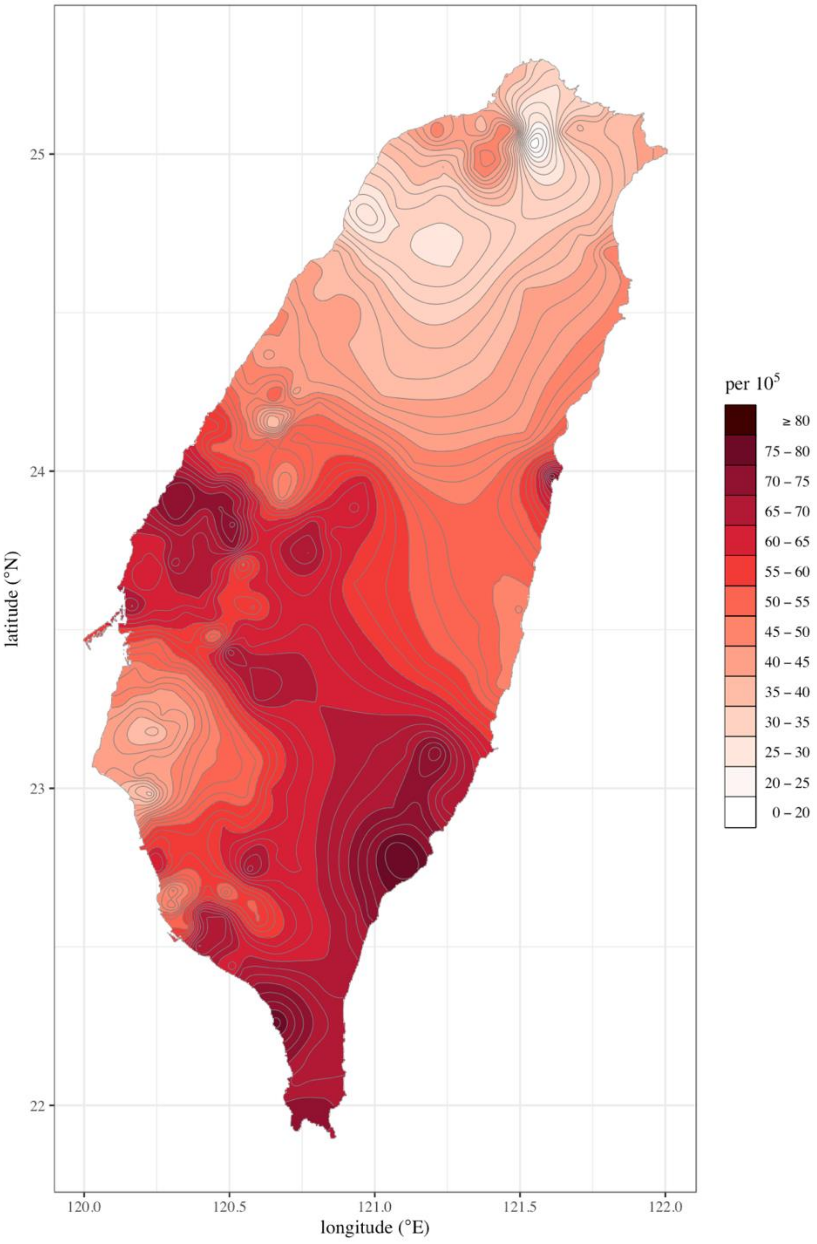

eFigure 3. Stabilized kriging analysis when the geometric centers of the two most populated regions (boroughs or villages) within a local administrative area were jointly representing the local administrative area

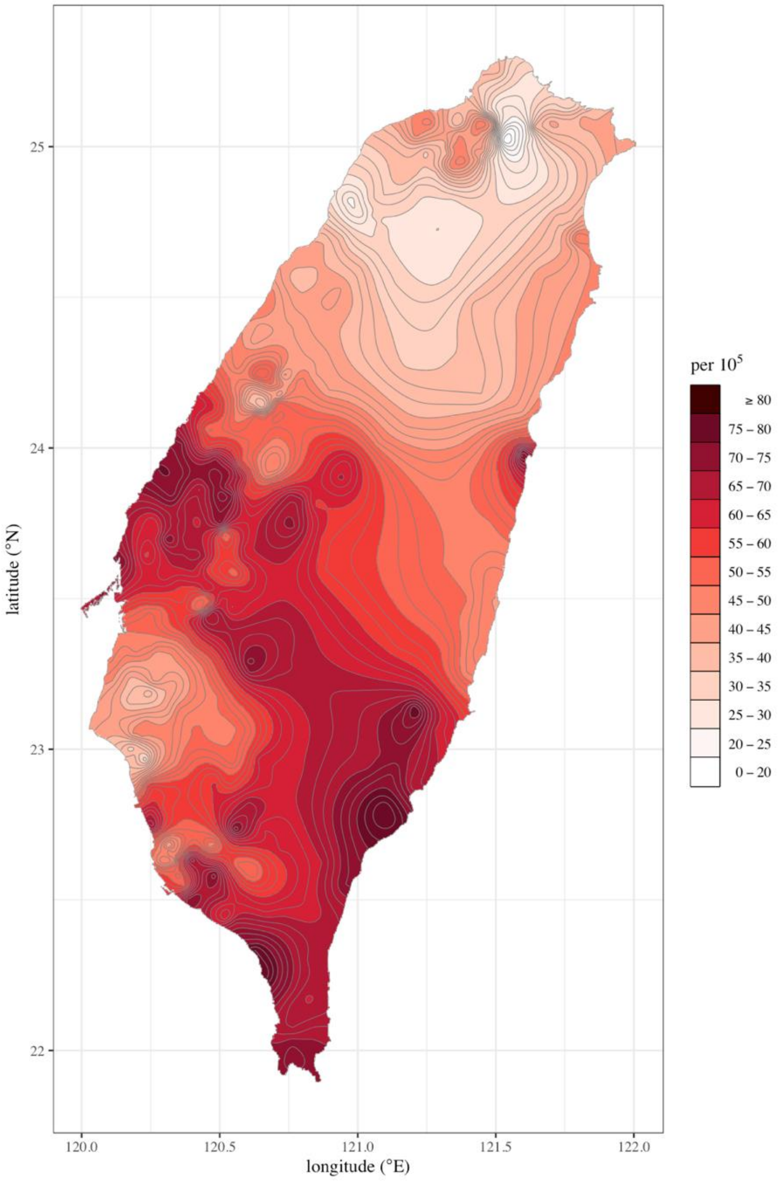

eFigure 4. Distances between the geometric and population centers of the 349 local administrative areas in the main island of Taiwan

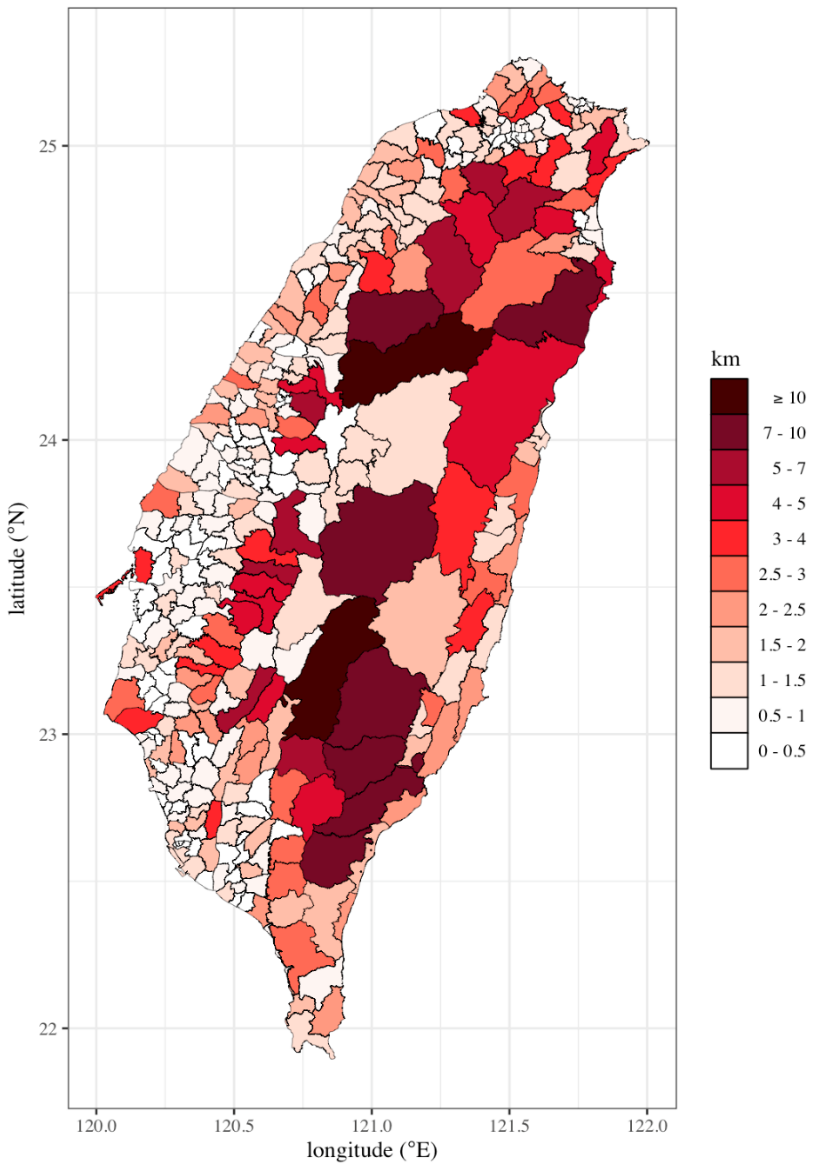

eFigure 5. Prevalence of betel nuts chewing in various counties and cities in the main island of Taiwan in 2017

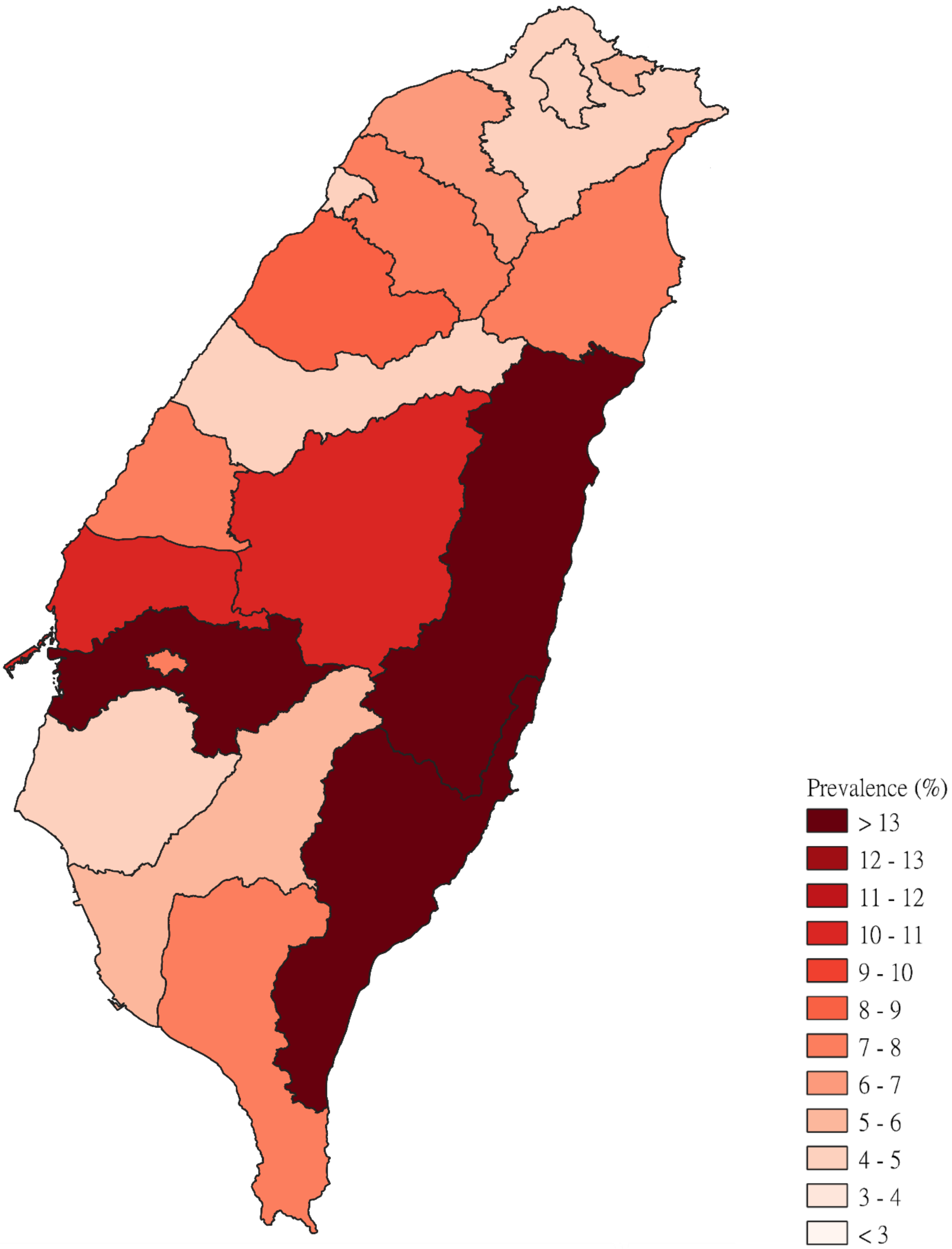

Supplement: Supplementary file 1 [file je-33-201-s001.pdf]
